# Vinyl Chloride: A Case Study of Data Suppression and Misrepresentation

**DOI:** 10.1289/ehp.7716

**Published:** 2005-03-24

**Authors:** Jennifer Beth Sass, Barry Castleman, David Wallinga

**Affiliations:** ^1^Natural Resources Defense Council, Washington, DC, USA; ^2^Environmental Consultant, Garrett Park, Maryland, USA; ^3^Institute for Agriculture and Trade Policy, Minneapolis, Minnesota, USA

**Keywords:** angiosarcoma, cancer, corporate, EPA, industry, IRIS, polyvinyl chloride, PVC, U.S. Environmental Protection Agency, vinyl chloride

## Abstract

When the U.S. Environmental Protection Agency (EPA) finalized its 2000 update of the toxicological effects of vinyl chloride (VC), it was concerned with two issues: the classification of VC as a carcinogen and the numerical estimate of its potency. In this commentary we describe how the U.S. EPA review of VC toxicology, which was drafted with substantial input from the chemical industry, weakened safeguards on both points. First, the assessment downplays risks from all cancer sites other than the liver. Second, the estimate of cancer potency was reduced 10-fold from values previously used for environmental decision making, a finding that reduces the cost and extent of pollution reduction and cleanup measures. We suggest that this assessment reflects discredited scientific practices and recommend that the U.S. EPA reverse its trend toward ever-increasing collaborations with the regulated industries when generating scientific reviews and risk assessments.

## Short History of Vinyl Chloride Regulation

Vinyl chloride (VC) is manufactured exclusively for polymerization into polyvinyl chloride (PVC), a plastic used in construction, packaging, electrical, and transportation industries; in household products such as flooring, water piping, videodiscs, and credit cards; and in medical products such as disposable intravenous bags, tubing, and bedpans. Global PVC production in 2002 was nearly 59 billion pounds (27 million metric tons), valued at approximately US$19 billion, with an average annual growth rate of 3% since 1997 (Linak and Yagi 2003). Approximately 15 billion pounds (7 million metric tons) of PVC was manufactured in the United States and Canada in 2002, primarily for domestic use (Linak and Yagi 2003). Pollution sources include production and fabrication, incineration, and landfills.

The first experimental evidence of VC carcinogenicity was reported in 1969 (Viola PL, unpublished data). Additional data were published in 1971 (Viola et al. 1971), followed in 1974–1975 by disclosure of rare liver cancers in workers (Creech and Johnson 1974; Creech and Makk 1975; Maltoni 1974, 1975; Maltoni et al. 1974). Upon release of these data, the U.S. Occupational Safety and Health Administration (OSHA) issued a notice effective April 1975 that VC and PVC production plants must reduce time-weighted average workplace exposure levels from 500 ppm to 1 ppm, to provide adequate worker protection (OSHA 1975).

When OSHA issued the new exposure limit of 1 ppm, industry spokespeople issued dire predictions of job loss and plant closures. However, in < 2 years virtually all U.S. manufacturing plants were able to meet the new standard while still maintaining rapid growth of sales volume. This was accomplished largely through better containment of unpolymerized VC monomer and improved exposure monitoring (OSHA 1975).

## Early Suppression of Evidence of Liver Damage

Industry leaders privately acknowledged that the existing limit of 500 ppm was excessive long before the OSHA standard (OSHA 1975). In 1959, internal industry experiments had revealed micropathology in rabbit livers after repeat exposures to 200 ppm VC monomer (Markowitz and Rosner 2002), causing Dow Chemical toxicologist V.K. Rowe (1959) to admit privately to his counterpart at B.F. Goodrich:

We feel quite confident … that 500 ppm is going to produce rather appreciable injury when inhaled 7 hours a day, five days a week, for an extended period. As you can appreciate, this opinion is not ready for dissemination yet and I would appreciate it if you would hold it in confidence but use it as you see fit in your own operations.

VC and PVC manufacturers also delayed public release of findings of liver angiosarcoma in VC-exposed rodents by Cesare Maltoni (Markowitz and Tosner 2002). In late 1972, the industry was briefed on Maltoni’s report of primary cancers of both liver and kidneys at exposures as low as 250 ppm, half the 500 ppm allowable exposure limit for workers. However, in a meeting with government officials 8 months later in the summer of 1973, industry representatives avoided any mention of Maltoni’s findings (Markowitz and Rosner 2002). The public learned of the deadly hazards of VC only in early 1974 through newspaper reports of the deaths of three workers in a B.F. Goodrich vinyl plant in Louisville, Kentucky (Creech and Johnson 1974). Like Maltoni’s experimental animals, the workers had liver angiosarcoma.

## Evidence of Nonliver Cancer

In addition to evidence of liver cancer, starting in the 1970s the industry’s own studies described excess cancers in nonliver sites, including the respiratory system and the brain (Tabershaw and Gaffey 1974). In a 1976 interoffice memo, Mitchell Zavon, a physician with Ethyl Corporation, acknowledged that

At present, the epidemiological work has amply demonstrated an association between high exposures to VCM [vinyl chloride monomer] and an increase in angiosarcoma of the liver, brain and lung tumors. (Zavon 1976)

A scientific review by the International Agency for Research on Cancer (IARC 1979) found that

Vinyl chloride is a human carcinogen. Its target organs are the liver, brain, lung and haemo-lymphopoietic system … there is no evidence that there is an exposure level below which no increased risk of cancer would occur in humans.

A second IARC review in 1987 supported the previous evaluation, citing more recent data that, in addition to angiosarcoma of the liver, VC caused hepatocellular carcinoma, brain tumors, lung tumors, and malignancies of the lymphatic and hematopoietic system (IARC 1987).

After the IARC evaluation, the industry commissioned British epidemiologist Richard Doll to review the previously published VC epidemiology. Doll combined data from four studies finding an aggregated excess risk of brain cancer [29 observed vs. 19.54 expected, standardized mortality ratio (SMR) = 148; confidence limits were not reported]; he reported this as “not statistically significant” and “nothing to suggest that they are occupational in origin” (Doll 1988). Doll (1988) downplayed risk of cancer in all sites other than liver, concluding that

[T]he mortality of the exposed men, other than that due to angiosarcoma of the liver, is typical of the normally healthy industrial worker—that is not to say that no other hazard exists, but that the effect of any other hazard is small.

Doll did not acknowledge funding sources in his article (Doll 1988), but in a legal deposition taken in a toxic tort case brought by a worker dying of brain cancer, Doll testified for the defendants that his 1988 report was conducted “on behalf of the Chemical Manufacturers Association” for which Doll received 12,000 British pounds (~ US$21,000) as “a donation to a charity in recompense” for his work (Doll 2000). The charity Doll selected was the Green College at Oxford, of which Doll is the founder and first warden (president).

Evidence of VC-associated brain cancer continued to accumulate after 1988. A 1991 Chemical Manufacturers Association (CMA)–sponsored follow-up study by Wong et al. (1991) reported significant excess deaths from cancer of the brain and central nervous system [23 observed vs. 12.76 expected death; SMR = 180; 95% confidence interval (CI), 114–271]. Wong et al. (1991) concluded that “this update confirms the excess in cancer of the brain and [central nervous system].” In addition, they reported significant excess deaths from cancer of the liver and biliary tract combined (37 observed vs. 6 expected deaths; SMR = 641; 95% CI, 450–884), from liver cancer excluding angiosarcoma (15 observed vs. 3.0 expected deaths; SMR = 500, significant at 1% level), and from biliary tract cancer excluding angiosarcoma (7 observed vs. 2.7 expected deaths; SMR = 259, significant at 5% level).

Two years later, in a highly unusual reversal, two of the original four authors published a retraction, saying “we conclude that our finding of an excess of brain cancer among U.S. vinyl chloride workers reported earlier was not likely related to the chemical” (Wong and Whorton 1993). The *Houston Chronicle* described the retraction and the uses made of it:

Wong hadn’t received permission from the study’s sponsor, the Chemical Manufacturers Association, to publish his data—data that could be used against the industry in lawsuits, that might alarm workers and attract regulators. The unauthorized publication provoked members of the CMA’s Vinyl Chloride Panel and touched off a months-long effort to persuade Wong to recant, documents show. Although Wong denies that he was pressured, he changed his story on vinyl chloride, declaring that the apparent excess of brain cancer deaths among workers might well be the result of “diagnostic bias”—better reporting and diagnosis of the disease in industry than in the general population …. Reprints of the Wong and Shah letters were distributed among the chemical companies and their attorneys. They are still cited by defendants in brain cancer cases, and are used to reassure workers about the safety of vinyl chloride and polyvinyl chloride. (Morris 1998)

In 2000, for the fourth time, an industry-sponsored study of VC epidemiology found an excess of brain cancer among exposed workers (Doll 1988; Mundt et al. 2000; Tabershaw and Gaffey 1974; Wong et al. 1991). Mundt et al. (2000) reported an increase in brain cancer among exposed workers (SMR = 142; 95% CI, 100–197), with mortality from brain cancer showing the largest excess for study subjects with the longest work history, based on 22 deaths (SMR = 177; 95% CI, 111–268). Nonetheless, Mundt et al. (2000) concluded that the “risk of mortality from brain cancer has attenuated, but its relationship with exposure to vinyl chloride remains unclear.”

## U.S. EPA Reassessment of VC Toxicology

Many of the U.S. Environmental Protection Agency (EPA) assessments of regulated chemicals are publicly available on its database, the Integrated Risk Information System (IRIS), which contains U.S. “EPA scientific consensus positions on potential human health effects from environmental contaminants” (U.S. EPA 1996). Although not a legal regulatory standard per se, such information is used by regulators at the state and federal level and by others worldwide in combination with exposure data to set cleanup standards and various exposure standards for air, water, soil, and food (Phibbs 2002). The widespread use of IRIS assessments is demonstrated by the fact that the database receives more than half a million visits monthly, from > 50 countries (IRIS 2005).

In 1994, the CMA’s Vinyl Chloride Panel initiated plans to work with the U.S. EPA on its IRIS assessment of VC. H.C. Shah, the industry panel manager, confirmed that the U.S. EPA “expressed an interest in working with industry to develop a scientifically-sound vinyl chloride risk assessment” (Shah 1994a, 1994b). At the meeting, CMA-sponsored scientists made presentations to the U.S. EPA on both the CMA-sponsored epidemiology and a prepublication risk model (Reitz and Gargas 1994; Shah 1994a, 1994b). The model, a physiologically based pharmacokinetic (PBPK) model, was designed to quantitatively express the relationship between external exposure to VC and internal dose at the liver, taking into account absorption, distribution, metabolism, and elimination of VC and its metabolites.

Although internal documents demonstrate that the U.S. EPA and the VC industry had been in joint discussions on an updated IRIS assessment of VC since 1994 (Shah 1994a, 1994b), it was not until 1996 that the U.S. EPA issued a public notice inviting submissions of technical information for VC and 10 other industrial chemicals to be assessed for the IRIS database (U.S. EPA 1996).

## U.S. EPA Standard Based on Overall Risk of Liver Cancer, Not Overall Cancer Risk

As noted above, as early as 1994 the VC industry had been promoting PBPK models for use by the U.S. EPA in its VC assessment. Two such models were presented to the U.S. EPA for its VC risk assessment. The models predicted that VC was 150-fold less (Reitz and Gargas 1994; Reitz et al. 1996) and 80-fold less (Clewell et al. 1995, 2001) potent as a carcinogen than values used at the time for environmental decision making, implying that pollution and cleanup standards could be weakened significantly. The final IRIS assessment relied on the Clewell model (Clewell et al. 1995, 2001), but with adjustments such that VC was estimated by the U.S. EPA to be 10-fold less potent as a carcinogen. Although the model was developed using only liver angiosarcoma tumor data, cancer estimates for the U.S. EPA assessment were revised to include all liver tumors but exclude all nonliver tumors (U.S. EPA 2000a). Because exposure was not adequately characterized in the epidemiology studies, the U.S. EPA cancer potency estimates were based on animal bioassay data.

Both models were designed to model only VC’s effects on the liver, despite scientific consensus that it is a multisite carcinogen in humans and experimental animals (Byren et al. 1976; Cooper 1981; Drew et al. 1983; Feron et al. 1979; Hagmar et al. 1990; IARC 1979, 1987; Infante 1981; Maltoni and Lefemine 1975; Maltoni et al. 1981; Monson et al. 1974; Mundt et al. 2000; Smulevich et al. 1988; Tabershaw and Gaffey 1974; Wagoner et al. 1980; Waxweiler et al. 1976, 1981; Weber et al. 1981; Wong and Whorton 1993; Wong et al. 1991; Wu et al. 1989).

VC administered orally or by inhalation to mice, rats, and hamsters produced tumors in the mammary gland (Feron et al. 1981; Hong et al. 1981; IARC 1987), leading Clewell et al. (1995) to suggest that

it seems reasonable that the evidence of increased mammary tumor incidence from VC should be considered at least qualitatively during risk management decisions regarding potential human VC exposure.

In its May 1999 draft VC assessment, the U.S. EPA had proposed to apply a protective 3-fold factor to adjust for VC’s possible induction of nonliver tumors (U.S. EPA 1999a). However, in a letter to the U.S. EPA, chemical manufacturers protested that

[T]he available epidemiological evidence does not support an association between vinyl chloride exposure and human cancer except angiosarcoma of the liver. The ill-advised three-fold uncertainty factor introduced by EPA to account for possible tumor induction at such sites can therefore be eliminated. (Price 1999)

In response, the U.S. EPA final VC assessment completely eliminated the protective factor it had originally included (U.S. EPA 2000a). In the same letter to the U.S. EPA, chemical manufacturers disputed the U.S. EPA statement that there is “suggestive epidemiological evidence that cancer of the brain, lung, and lymphopoietic system are associated with exposure,” saying it “should be deleted from the final review” (Price 1999). The U.S. EPA complied (U.S. EPA 2000a).

The U.S. EPA assessment’s exclusion of risks to organs other than liver is striking. The U.S. EPA justifies this approach on two grounds: first, relying on the conclusions of Richard Doll that evidence for induction of nonliver tumors is weak (Doll 1988); and second, suggesting that the liver is the most sensitive end point and therefore regulatory standards protective of liver cancer would adequately protect all other sites from cancer risk (U.S. EPA 2000b). However, this limited view precludes the U.S. EPA from developing a standard based on an assessment of the total cancer risk to all organs from VC exposure, as required by U.S. EPA guidelines for calculating carcinogenic risk (U.S. EPA 1999b, 2005).

Downplaying risk to nonliver cancer sites leaves the public and exposed workers inadequately informed of the health threat posed by exposure to VC-containing products, processes, and pollution. Medical professionals are less likely to suspect a link to VC exposures in patients with nonliver cancers, and thus causal links are more likely to be overlooked. Downplaying of nonliver cancer risks by the U.S. EPA may also have important implications in litigation of compensation cases, because claims for cancers at sites other than the liver are vigorously disputed in the courts.

## Peer Review Reflects Industry Participation

The U.S. EPA’s external peer review process is intended to ensure that a scientifically credible assessment is produced. However, at least 7 of the 19 external peer reviewers of the VC assessment were chemical industry employees and consultants, 4 were government representatives, and none represented unions or public interest groups (U.S. EPA 2000b). This committee accepted the assertion by the U.S. EPA that human exposure limits based on liver cancer would be sufficiently protective against cancer developing in other tissues. The committee rejected the use of any protective adjustment factor to account for the possibility of nonliver cancer risk (U.S. EPA 2000aa). As noted above, the final assessment made no adjustments for the possibility of cancer at nonliver sites.

The final VC assessment currently posted on the IRIS database (U.S. EPA 2000b) assigns a cancer risk from VC inhalation (8.8 × 10^−6^ risk per μg/m^3^; an excess of 8.8 cases per 1 million people exposed over a lifetime to an average of 1 μg/m^3^ VC) that is about 10-fold lower than the previous assessment (8.4 × 10^−5^ risk per μg/m^3^; an excess of 84 cases per 1 million people exposed over a lifetime to an average of 1 μg/m^3^ VC). As a result, allowable pollution levels may increase by 10-fold.

## The Trend to Incorporate Industry Participation in U.S. EPA Scientific Assessments

For some of the most widespread and toxic chemicals under regulation, the manufacturers are generating much of the data (often unpublished) used for risk assessment and are working closely with the U.S. EPA to evaluate available data and produce risk assessments. Unfortunately, the efforts of the regulated industries often outweigh the ability of the public, unions, and public interest groups to participate in developing regulations. In a 2002 interview, Paul Gilman, at that time the science adviser to U.S. EPA Administrator Whitman, expressed dissatisfaction with the industry submissions for IRIS:

[I]t is taking staff as much or more time to work with the outside parties as it does to develop in-house toxicological reviews, Gilman said. To date, the process has not saved the time or resources it was designed to save. (Phibbs 2002)

Nonetheless, in late August 2004, the U.S. EPA announced changes to its pesticide review process “that would give industry officials greater input in the science behind its risk reviews … in an effort to reduce the agency’s review times” (Inside EPA 2004). The trend toward increasing industry participation allows corporate interests with products under regulation to more effectively recommend acceptable limits of public exposure to their own products and wastes, while placing an unrealistic burden on the U.S. EPA scientists and the public to provide adequate peer review and oversight. Public confidence is undermined when commercial interests, instead of scientific evaluations, shape public health policy.
